# The positive impact of robotic‐assisted BCR TKA on post‐operative joint line restoration, lateral posterior condylar offset and standard and advanced activity in the 2011 Knee Society Score than the conventional jig‐based technique

**DOI:** 10.1002/jeo2.70288

**Published:** 2025-06-01

**Authors:** Takao Kaneko, Kosuke Shiga, Ayakane Yamamoto, Kazuki Amemiya, Masaru Omata, Shu Yoshizawa

**Affiliations:** ^1^ Ichinomiya Onsen Hospital Adult Reconstruction Center Fuefuki‐city Yamanashi Japan; ^2^ Department of Rehabilitation Ichinomiya Onsen Hospital Fuefuki‐city Yamanashi Japan

**Keywords:** bi‐cruciate retaining total knee arthroplasty, conventional jig‐based knee arthroplasty, patient‐reported outcome measurements, robotic‐assisted total knee arthroplasty, three‐dimensional computed tomography

## Abstract

**Purpose:**

The aim of this study was to compare post‐operative short‐term patient‐reported outcome measurements (PROMs) between robotic‐assisted (RA) and the conventional jig‐based technique using bi‐cruciate retaining total knee arthroplasty (BCR TKA).

**Methods:**

This retrospective single‐surgeon consecutive cohort analysis compares 33 RA‐BCR TKA patients (Robot group) to 32 conventional TKA patients (Conventional group). Lateral distal femoral angle (LDFA), medial proximal tibial angle (MPTA), femoral and tibial component rotational alignment, distal and posterior femoral osteotomy (mm) were compared between the two groups using three‐dimensional computed tomography (3DCT) measurements. 2011 Knee Society Score (KSS), Western Ontario and McMaster Universities Arthritis Index (WOMAC), Forgotten Joint Score and Patella score were collected more than 1 year after surgery.

**Results:**

Pre‐operative knee scores were significantly higher in the Robot group (mean: 20.5) than in the Conventional group (mean: 16.8), while functional scores were significantly lower in the Robot group (mean: 57.7) than in the Conventional group (mean: 69.6). The Robot group showed significantly greater improvements in post‐operative extension angle (*p* < 0.01), and standard and advanced activity in the 2011 KSS (*p* < 0.01). LDFA, MPTA and rotational alignment of the femoral and tibial components showed no significant difference between the two groups. Medial distal femoral osteotomy (mm) was significantly greater in the Conventional group (*p* < 0.01). Lateral posterior femoral osteotomy (mm) was significantly greater in the Robot group (*p* < 0.01). The robotic group had significantly more cases of joint line restoration and reduced lateral posterior condylar offset than the Conventional group.

**Conclusions:**

RA‐BCR TKA improved post‐operative extension angle and standard and advanced activity in the 2011 KSS more than the Conventional TKA.

**Level of Evidence:**

Level III.

**Abbreviations**
3DCT: three‐dimensional computed tomography
ACL: anterior cruciate ligament
AP: anterior‐posterior
BCR: bi‐cruciate retaining
BCS: bi‐cruciate stabilized
CR: cruciate retaining
E/R: external rotation
FJS‐12: forgotten joint score
FTA: femoro‐tibial angle
I/R: internal rotation
KSS: knee society score
LDFA: lateral distal femoral angle
LJG: lateral joint gap
MA: mechanical axis
MJG: medial joint gap
MPTA: medial proximal tibial angle
OA: osteoarthritis
PCO: posterior condylar offset
PROM: patient‐reported outcome measurement
PS: posterior stabilized
PTA: posterior tilt angle
RA: robotic‐assisted
ROM: range of motion
TKA: total knee arthroplasty
WOMAC: Western Ontario and McMaster Universities Arthritis Index

## INTRODUCTION

Total knee arthroplasty (TKA) is the gold standard treatment of late‐stage osteoarthritis (OA); however, approximately 20% of patients are not satisfied with their surgically restored knees [4, 9, 33].

The majority of current TKA designs sacrifice the anterior cruciate ligament (ACL) without substituting for its function. The loss of the ACL function has significant effects on the kinematics of the knee [10]. Recently, bi‐cruciate retaining (BCR) TKA has been attracting attention again. Fluoroscopic imaging has demonstrated contact points in full extension, as well as posterior rollback at 90° flexion that more closely replicates the normal knee [40]. However, the first‐generation BCR TKAs, namely the polycentric knee [18] and geometric knee [12], are complicated techniques that cause what is known as a ‘kinematic conflict (KL)’, where ligamentous tension increases at certain flexion angles due to the preservation of all ligaments, resulting in early post‐operative revision [36, 42].

Recently, novel robotic‐assisted (RA) technologies have been introduced to the field of TKA with the promise of improving component alignment by targeting precision in bone preparation and soft tissue balancing. As an example, handheld image‐free RA surgical systems (NAVIO® and CORI®, Robotic surgical system, Smith & Nephew, Inc.) make it possible to intra‐operatively determine component alignment and the amount of damaged osteochondral cartilage to be removed while considering soft tissue balancing over the full range of motion (ROM) [26, 27, 28]. The second‐generation Journey II XR (Smith& Nephew, Inc.) with an oblique three‐angle femorotibial joint line and the asymmetrical joint surface design was used. Despite numerous studies evaluating outcomes of image‐based RA‐TKA, and some comparing radiographic and early outcomes following imageless RA bi‐cruciate stabilized (BCS) TKA, no previous studies have compared patient‐reported outcome measurements (PROMs) after TKA with the BCR prosthesis using RA technology versus the conventional jig‐based technique.

The aims of this study were (1) to compare post‐operative short‐term PROMs following the RA technique versus the conventional jig‐based technique using XR prosthesis, and (2) to investigate soft tissue balance, osteotomy (mm) and component alignment by RA technique to improve PROMs without kinematic conflicts.

Our hypothesis was that RA‐BCR TKA would improve post‐operative PROMs than the conventional jig‐based technique.

## MATERIALS AND METHODS

### Participants and study subjects

**Figure 1:**
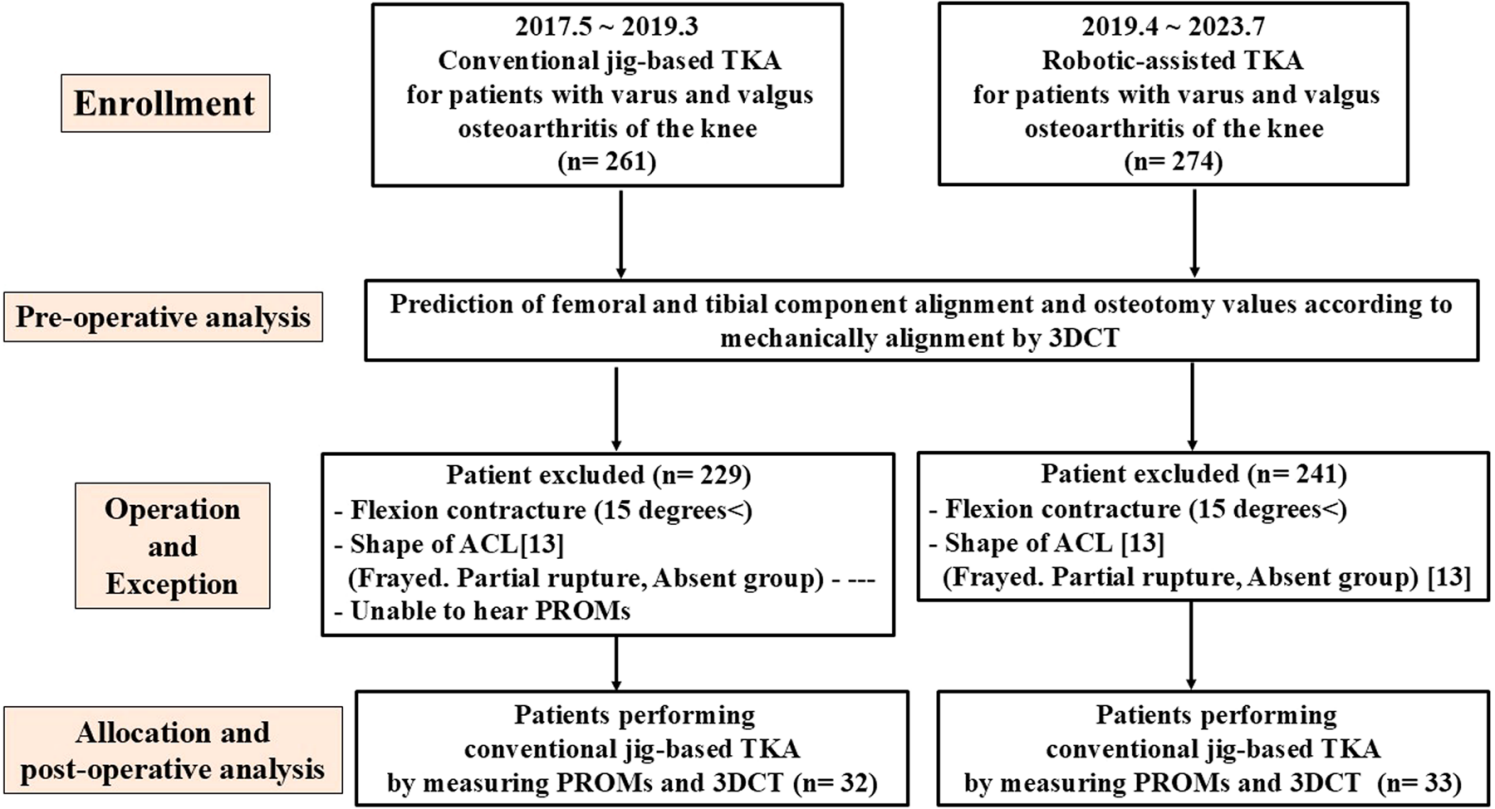
Patients flow diagram including patients. 3DCT, three‐dimensional computed tomography; ACL, anterior cruciate ligament; PROM, patient‐reported outcome measurement; TKA, total knee arthroplasty.

The study design was approved by the Ethics Review Committee (21013). All patients who participated provided written informed consent. Between May 2017 and March 2019, 261 conventional jig‐based (Conventional group) TKAs and between April 2019 and July 2023, 274 RA TKAs (Robot group) were performed (Figure 1). The surgical indications for BCR TKAs were knee primary OA or osteonecrosis of more than two compartments, intact cruciate and collateral ligaments and ACL assessment was conducted intraoperatively to evaluate its integrity. The exclusion criteria were severe fixed flexion contractures with more than 15° of flexion and specific ACL abnormalities (fraying, rupture, or partial or absent ligament) (Figure 1) [41]. In addition, complete data are available for a minimum follow‐up of 1 year. In this study, 32 conventional jig‐based BCR TKAs and 33 RA‐BCR TKAs met the following inclusion and exclusion criteria (Figure 1). BCS prosthesis was used for patients excluded from both groups. As a consecutive series for both groups, all procedures were performed by a single surgeon.

### Surgical procedure

Conventional TKAs were performed using conventional manual surgical procedures with the measured resection technique. After inflating a tourniquet to 300 mmHg at the beginning of the procedure, a subvastus arthrotomy was performed. A distal femoral osteotomy was performed at the valgus angle of the femur; this angle was measured between the mechanical axis (MA) and the anatomical axis using an intramedullary resection guide during pre‐operative three‐dimensional computed tomography (3DCT) planning for the entire lower extremity. The rotational angle of the femoral component was aligned parallel to the surgical epicondylar axis and perpendicular to the Whiteside line. An extramedullary resection guide was used for proximal tibial osteotomy. The angle of the osteotomy was perpendicular to the MA, and the physiological tibial posterior tilt in each patient was decided via pre‐operative 3DCT planning for the entire lower extremity [44]. The landmark used to determine the rotational alignment of the tibia was Akagi's line, defined as a straight line from the middle of the posterior cruciate ligament to the medial border of the patellar tendon attachment site [1, 2]. We managed the soft tissue releases to obtain good balance.

A different surgical approach was used for the RA group. After inflating a tourniquet to 300 mmHg at the beginning of the procedure, a medial parapatellar arthrotomy was performed. Surfaces were ‘painted’ with an optical probe to conduct landmark registration, assess ROM and perform mapping of varus‐valgus laxity, femoral condyle anatomy, and the ACL footprint and tibial plateau. A virtual model of the knee was thus created.

The MA was set as the target alignment using a pre‐operative 3DCT‐based system. The planning for prosthesis size, component alignment, and osteochondral resection volume was modified based on the soft tissue balancing of each patient over the full ROM using an intra‐operative joint‐balancing procedure.

Arthritic cartilage and bone were methodically removed with a handheld sculptor using the NAVIO® (16 knees) and CORI® (17 knees) RA techniques. The sculptor could be used to burr away arthritic cartilage and bone at the distal femur and proximal tibia. RA technique continuously tracked the position of the patient's lower limb and the progress of bone resection using a navigation system camera.

### Pre‐ and post‐operative 3DCT images

**Figure 2:**
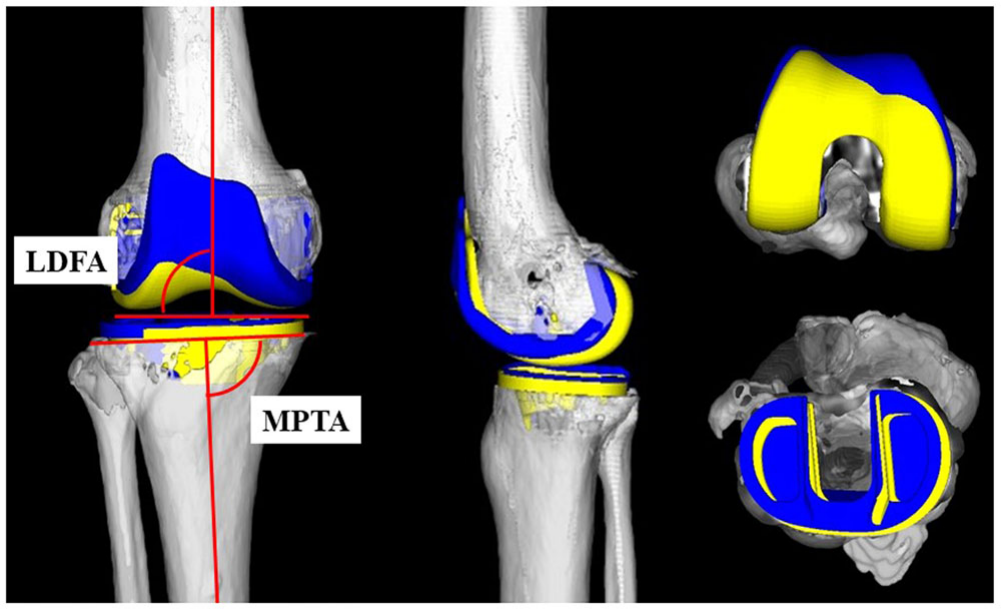
Pre‐operative plan three‐dimensional computed tomography (3DCT) plan and post‐operative 3DCT images were shown. Three‐dimensional computer‐aided design data of femoral and tibial components were fit to the 3DCT plan and images. Blue components (preoperative plan image), Yellow components (post‐operative component). LDFA, lateral distal femoral angle; MPTA, medial proximal tibial angle.

In both groups, pre‐operative plans in all cases were developed using 3DCT data (Aquilion Start TSX‐037A; Canon Medical Systems Co., Ltd.) of the relevant extremities. Post‐operative CT scans were obtained 4 weeks after surgery. 3D data of femoral and tibial components were overlaid on post‐operative 3DCT images (Figure 2). Post‐operative 3DCT images of the femur and tibia were superimposed onto those of the pre‐operative 3DCT plan using computer software program (ZedView, ZedKnee; LEXI Co., Ltd.) (Figure 2) [26, 27, 28].

**Figure 3:**
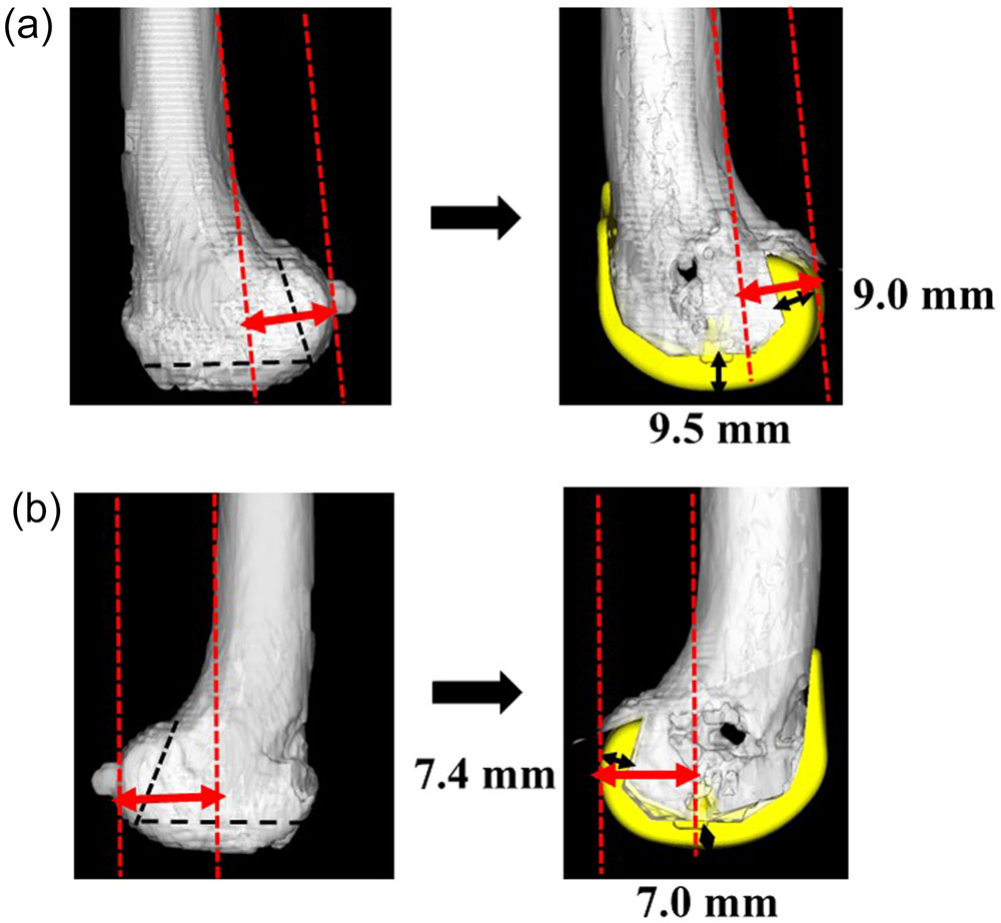
(a) Pre‐operative plan three‐dimensional computed tomography (3DCT) plan and post‐operative images 3DCT image were shown. We measured medial distal and posterior femoral osteotomy volumes. The distal and posterior thicknesses in the medial femoral condyle of the femoral component in Journey Ⅱ XR are 9.5 and 9.0 mm. Robot group: pre‐ and post‐operative PCO decrease: 21 knees, increase: 6 knees, constant: 0 knees. Conventional group: pre‐ and post‐operative PCO decrease: 21 knees, increase: 10 knees, constant: 1 knee. Black arrows: osteotomy line, red arrows: posterior condylar offset (PCO). (b) Pre‐ operative plan 3DCT plan and post‐operative images 3DCT image were shown. We measured lateral distal and posterior femoral osteotomy volumes. The distal and posterior thicknesses in the lateral femoral condyle of the femoral component in Journey Ⅱ XR are 7.0 and 7.4 mm. Robot group: pre‐ and post‐operative PCO decrease: 20 knees, increase: 7 knees, constant: 0 knees. Conventional group: pre‐ and post‐operative PCO decrease: 3 knees, increase: 28 knees, constant: 1 knee. Black arrows: osteotomy line, red arrows: PCO.

Lateral distal femoral angle (LDFA), medial proximal tibial angle (MPTA) (Figure 2), femoral distal and posterior femoral osteotomy (mm) (Figure 3), and joint line height (+: elevation, −: descent, relative to the distal thickness of the prosthesis) were measured using 3DCT images. Moreover, 3DCT images were used to measure RA of the femur (+: external rotation; E/R, −: internal rotation; I/R) was defined as the angle between the line perpendicular to the surgical epicondylar axis and the line between the lowest point of the medial epicondyle and the midpoint of the lateral epicondyle [7] and measure RA of the tibia relative to Akagi's line (−: E/R, +: I/R) [1], the correction angle relative to the tibial physiological posterior tilt angle (PTA) of the individual patient (+: post‐operative PTA greater than pre‐operative, −: post‐operative PTA smaller than pre‐operative). The femoro‐tibial angle (FTA) was also measured using 3DCT images.

Inter‐class correlation coefficients for 3DCT evaluations in the coronal, sagittal and axial planes were 0.901, 0.899 and 0.881 for the femur, and 0.924, 0.911 and 0.899 for the tibia, respectively. Intra‐class correlation coefficients for the coronal, sagittal and axial planes were 0.956, 0.903 and 0.878 for the femur, and 0.918, 0.815 and 0.896 for the tibia, respectively [26, 27, 28].

### PROMs

Knee Society Scores (KSS: Knee score and Function score [21]) were measured before surgery. Four patient‐reported subscales of the 2011 KSS (symptoms, patient satisfaction, patient expectations, standard and advanced activities) [39], three patient‐reported subscales of the Western Ontario and McMaster Universities Arthritis Index (WOMAC) (pain, stiffness and physical function) [6], 12 questions in the Forgotten Joint Score (FJS‐12) [5], and four patient‐reported subscales of the Patella score (anterior knee pain, quadriceps strength, ability to rise from a chair and stair‐climbing) [16] were measured at a mean of 20.3 months (ranges, 12–48 months, 12–24 months: 27 knees, 25–36 months: 3 knees, 37–48 months: 3 knees) post‐operatively in the Robot group and 20.5 months (ranges, 12–67 months, 12–24 months: 26 knees, 25–36 months: 4 knees, 37–48 months: 1 knee, 67 months: 1 knee) post‐operatively in the Conventional group (Table 1).

**Table 1:** Pre‐operative patient demographics data.

	Robot group (*N* = 33)	Conv group (*N* = 32)	*p* **value**
Age (year)	74.1 (SD 6.8; 60–85)	77.1 (SD 5.6; 65–86)	n.s
Gender (male:female)	5:27	8:24	
BMI (kg/m^2^)	24.4 (SD 2.7; 18.7–30)	24.1 (SD 2.3; 19.2–27.2)	n.s
Extension angle (°)	−9.3 (SD 4.3; −15 to 0)	−11.7 (SD 5.4; −15 to 0)	n.s
Flexion angle (°)	123.3 (SD 4.4; 114–130)	120.9 (SD 8.3; 100–145)	n.s
FTA angle (°)	179.9 (SD 3.1; 173–185)	180.5 (SD 2.9; 177–185)	n.s
Knee score	20.5 (SD 6.5; 4–31)	16.8 (SD 9.5; 3–32)	<0.01
Function score	57.7 (SD 16.5; 20–80)	69.6 (SD 11.5; 35–80)	<0.01
F/U period (months)	20.3 (SD 11.4; 12–48)	20.5 (SD 11.5; 12–67)	n.s

*Note*: Mean, SD and range were provided

Abbreviations: BMI, body mass index; Conv, conventional; F/U, follow‐up; FTA, femoro‐tibial angle; n.s, not significant; SD, standard deviation.

### Statistical analysis

Means and standard deviations (SDs) are used to describe the data. All statistical analyses were performed with SPSS version 24.0 software (SPSS Inc.). Pre‐operative factors, post‐operative PROMs and 3DCT measurements, which were parameters between the two groups, were validated using the Mann‐Whitney *U* test. Statistical significance was set at a *p* value < 0.05, and the 95% confidence interval was measured.

The estimated sample size was 48 patients to compare the clinical outcomes between the RA and Conventional groups according to the statistical power analysis using G*Power 3 (Heinrich Heine Universität Düsseldorf, FRG) [15]. Thus, we used 60 knees to perform this study. All significance tests were two‐tailed, and a significance level of *p* < 0.05 was used for all tests.

## RESULTS

Pre‐operative variables, including age, gender, ROM, FTA, knee score, function score and follow‐up period, are shown for both groups in Table 1. Pre‐operative knee scores were significantly higher in the Robot group (mean: 20.5) than in the Conventional group (mean: 16.8), while functional scores were significantly lower in the Robot group (mean: 57.7) than in the Conventional group (mean: 69.6) (Table 1). Post‐operative ROM and PROMs are shown in Table 2.

**Table 2:** Post‐operative range of motion, post‐operative 2011 Knee Society Score, Western Ontario and McMaster Universities Osteoarthritis Index (WOMAC) and Patella score.

	Robot group (*N* = 33)	Conv group (*N* = 32)	*p* **value**
Extension angle (°)	0.3 (SD 2.5; −5 to 2.0)	−2.5 (SD 5.4; −10 to 0)	<0.01
Flexion angle (°)	124.9 (SD 11.3; 95–147)	122.1 (SD 15.1; 85–145)	n.s
2011 Knee Society Score			
Symptom (25)	19.8 (SD 4.1; 8–25)	19.3 (SD 4.9; 7–25)	n.s
Patient satisfaction (40)	26.4 (SD 6.8; 16–40)	25.3 (SD 8.0; 12–40)	n.s
Patient expectation (15)	9.7 (SD 2.7; 4–15)	9.3 (SD 2.8; 4–15)	n.s
Advanced activity (100)	71.9 (SD 9.2; 55–93)	62.6 (SD 15.8; 37–91)	<0.01
WOMAC score			
Pain (20)	15.9 (SD 3.8; 6–20)	15.4 (SD 4.1; 6–20)	n.s
Stiffness (8)	5.2 (SD 2.7; 0–8)	5.7 (SD 2.3; 1–8)	n.s
Daily activities (68)	39.2 (SD 12.1; 28–68)	42.5 (SD 14.3; 18–67)	n.s
FJS‐12 (100)	57.0 (SD 21.5; 21–100)	55.3 (SD 18.9; 17–92)	n.s
Patella score			
Anterior knee pain (15)	12.7 (SD 3.3; 5–15)	11.3 (SD 5.2; 0–15)	n.s
Quadriceps strength (5)	4.4 (SD 1.0; 1–5)	3.4 (SD 1.3; 1–5)	n.s
Ability to rise from chair (5)	3.9 (SD 1.0; 3–5)	3.4 (SD 1.4; 1–5)	n.s
Stair‐climbing (5)	3.1 (SD 1.0; 2–5)	3.4 (SD 1.1; 2–5)	n.s

*Note*: Mean, SD and range were provided.

Abbreviations: Conv, conventional; FJS‐12, forgotten joint score 12; SD, standard deviation.

Compared to the Conventional group, the Robot group had significantly better post‐operative extension angles (*p* < 0.01) and standard and advanced activity in the 2011 KSS (*p* < 0.01) (Table 2). In these results, the differences proved to be no more than the minimal clinically important difference (MCID) of each PROM (Table 3). Medial distal femoral osteotomy (mm) in the Conventional group was significantly greater than that in the Robot group (*p* < 0.01). The results showed that pre‐operative joint line height was maintained post‐operatively in many cases in the Robot group (*p* < 0.01) (Table 4). Lateral posterior femoral osteotomy (mm) was significantly greater in the Robot group than in the Conventional group (*p* < 0.01) (Table 4). LDFA, rotational alignment of the femoral and tibial component, and MPTA showed no significant difference between the two groups. The most common type of LDFA in the Robot group was varus alignment, while the most common type in the Conventional group was valgus alignment. The rotational alignment of the femoral component was less than 3° in 15 knees for the Robot group, but only 6 knees for the Conventional group. Tibial rotational alignment was more I/R alignment in both groups and with regard to MPTA, varus alignment was the most common in both groups (Table 4). The correction angle relative to the physiological PTA of tibia showed no significant difference between the two groups.

**Table 3:** Minimal clinically important difference (MCID) based on distribution‐based method.

	Mean	SD	SEM	95% CI	AUC	*p* **value**	Sensitivity (%)	Specificity (%)	MCID
2011 KSS									
Symptom (25)	19.48	4.56	0.58	(18.33–20.64)	0.54	*p* < 0.001	14.29	81.82	18.35
Patient satisfaction (40)	25.77	7.25	0.92	(23.95–27.60)	0.52	*p* < 0.001	11.43	93.94	23.97
Patient expectation (15)	9.61	2.8	0.36	(8.91–10.32)	0.52	*p* < 0.001	11.43	100	8.92
Advanced activity (100)	67.56	13.6	1.73	(64.14–70.98)	0.43	*p* < 0.001	2.86	100	64.18
WOMAC									
Pain (20)	15.71	3.94	0.51	(14.69–16.73)	0.54	*p* < 0.001	11.43	77.78	14.73
Stiffness (8)	5.48	2.51	0.31	(4.87–6.09)	0.52	*p* < 0.001	0	96.3	4.86
Daily activity (68)	40.61	12.89	1.62	(37.43–43.79)	0.52	*p* < 0.001	0	96.3	37.4
FJS‐12 (100)	56.11	19.31	2.4	(51.37–60.85)	0.52	*p* < 0.001	0	96.3	51.31
Patella score (30)	22.95	5.53	0.73	(21.52–24.39)	0.54	*p* < 0.001	8.57	88.89	21.58
Anterior knee pain (15)	12.02	4.39	0.55	(10.91–13.12)	0.53	*p* < 0.001	68.57	51.85	10.92
Quadriceps strength (5)	3.92	1.31	0.17	(3.58–4.26)	0.51	*p* < 0.001	57.14	48.15	3.59
Ability to rise from chair (5)	3.68	1.25	0.16	(3.37–3.98)	0.53	*p* < 0.001	48.57	66.67	3.37
Stair‐climbing (5)	3.26	1.1	0.14	(2.98–3.54)	0.54	*p* < 0.001	11.43	81.48	2.98

*Note*: Mean, standard deviation (SD), standard error of measurement (SEM), confidence interval (CI) and area under the curve (AUC) were provided.

Abbreviations: Conv, conventional; FJS‐12, forgotten joint score 12; WOMAC, western ontario and mcmaster universities osteoarthritis index.

**Table 4:** Coronal alignment and rotational angle of femoral and tibial components, amount of distal, posterior femoral osteotomy using post‐operative 3DCT image.

	Robot group (*N* = 33)	Conv group (*N* = 32)	*p* **value**
Lateral distal femoral angle (°), cases	90.3 (SD 1.9; 87–94) Varus: 15, neutral: 4, valgus: 14	89.8 (SD 1.7; 86.5–94.1) Varus: 11, neutral: 7, valgus: 14	n.s
Medial distal femoral osteotomy (mm)	8.5 (SD 1.7; 4.5–12.2)	10.1 (SD 3.4; 6.0–20.3)	<0.01
Lateral distal femoral osteotomy (mm)	6.6 (SD 2.3; 1.4–11.5)	8.1 (SD 3.4; 2.9–17.4)	n.s
Joint line height (mm)	−0.9 (SD 1.7; −5 to 2.6)	0.7 (SD 3.4; −5.9 to 10.8)	*<*0.01
Femoral component rotational angle (°), cases	3.0 (SD 2.4; −1.2 to 5.3), less than 3°: 15	4.5 (SD 2.2; −1.2 to 7.5) less than 3°: 6	n.s
Medial posterior femoral osteotomy (mm)	11.0 (SD 2.2; 6.5–15.1)	8.7 (SD 5.7; 7.1–11.6)	n.s
Lateral posterior femoral osteotomy (mm)	8.7 (SD 2.5; 2.1–12.6)	5.7 (SD 1.5; 3.2–8.7)	*<*0.01
Medial tibial proximal angle (°), cases	89.5 (SD1.7; 85.5–94) Varus: 20, neutral: 7, valgus: 6	89.3 (SD1.7; 85–94) Varus: 15, neutral: 14, valgus: 3	n.s
Tibial component rotational angle (°)	7.3 (SD 7.1; −1.0 to 20) I/R: 29, E/R: 4	4.4 (SD 6.4; −11.5 to 19) I/R: 26, E/R: 6	n.s
Tibial posterior tilt correction value	−0.8 (SD 1.8; −6 to 1.5)	−0.3 (SD 2.1; −6 to 2.5)	n.s

*Note*: Mean, SD and range were provided. A positive value represents E/R, and a negative value represents I/R of the femoral components. A positive value represents I/R, and a negative value represents E/R of the tibial components.

Abbreviations: 3DCT, three‐dimensional computed tomography; Conv, conventional; E/R, external rotation; I/R, internal rotation; ns, not significant; SD, standard deviation.

The intra‐operative medial joint gap (MJG) and lateral joint gap (LJG) in extension and at 90° of flexion are shown in Table 5. No cases of complications such as revision surgery were observed between the two groups.

**Table 5:** The mean post‐operative knee flexion angle after first‐ and second‐generation bi‐cruciate retaining prostheses.

Post‐operative flexion angle	Prosthesis	Reference
102	Hermes 2C	Jenney and Jenny 1998 [23]
103	Hermes 2C	Sabouret et al. 2013 [37]
104	N2C	Moro‐oka et al. 2007 [32]
107	Hermes 2C	Cloutier et al. 1999 [11]
110	Anatomical total knee	Townley et al. 1985 [43]
119	Biopro	Pritchett 2011 [35]
117	Journey II XR	Kono et al. 2019 [30]
127	Journey II XR	Inui et al. 2023 [22]
124.9	RA Journey II XR	In this study
122.1	Conv Journey II XR	

Abbreviations: Conv, conventional; RA, robotic‐assisted.

## DISCUSSION

The most important findings of this study were that (1) RA technique improved post‐operative extension angle and standard and advanced activity in 2011 KSS compared to the conventional jig‐based technique, (2) post‐operative 3DCT measurements in the Robot group showed that the joint line was not elevated and (3) lateral posterior femoral osteotomy (mm) was significantly greater than the Conventional group.

We have previously reported that achieving the slightly laxity MJG from 0° to 140° of flexion, the tightness of the LJG at 90° of flexion and a physiological laxity (MJG < LJG) from 0° to 140° of flexion improved post‐op PROMs when utilizing Journey Ⅱ XR with a balancer [25]. Therefore, in the RA surgical technique, component alignment and osteotomy (mm) were determined by considering the soft tissue balancing with reference to these.

The XR prosthesis is an anatomical system with unique design features, including the thickness of distal and posterior condyle in femoral component difference from left to right (Figure 3).

XR prosthesis was selected intra‐operatively in only 12.2% (32/261) of the Conventional group and 12.0% (33/274) of the Robot group (Figure 1).

In the Robot group, pre‐operative Knee score was better than in the Conventional group, yet pre‐operative functional score was worse, while post‐operative standard and advanced activity in the 2011 KSS was significantly better than in the Conventional group. Factors contributing to these results include the possibility of obtaining good soft tissue balancing prior to bone resection with the introduction of RA [31]. Recent data have suggested that variability in the component alignment is well tolerated as long as the soft tissues are balanced using RA [8, 20], and we also revealed that the post‐operative phenotypic differences in the coronal plane proposed by Hirshmann et al. [19] did not affect post‐operative PROMs after RA BCS TKA [28].

In this study, unlike the Conventional group, the Robot group was able to obtain a good extension angle and joint line restoration. The osteotomy (mm) did not exceed the thickness of the distal femoral medial implant (9.5 mm), which prevented overstrain of both cruciate ligaments and had a positive effect on the PROMs than the Conventional group.

The alignment of femoral components showed no significant difference between the two groups. On the other hand, there were more cases (15 cases) of post‐operative femoral component varus alignment with the increase in MJG at extension than the Conventional group (Table 4). The results suggest that there was no significant difference in lateral distal femoral osteotomy volume between the two groups (Table 4).

The rotational alignment of the femoral component showed no significant difference between the two groups, but there were more cases (15 cases) with a rotational alignment of the femoral component of less than 3° in the Robot group than in the Conventional group (6 cases). Due to decreased MJG (or increased LJG at 90°) in the Robot group, the lateral posterior femoral osteotomy volumes were significantly greater than those of the Conventional group. There was a reduction in lateral posterior condylar offset (PCO) post‐operatively compared to pre‐operatively in 26 knees in the Robot group compared to 3 knees in the Conventional group (Figure 3).

Conversely, there was a positive correlation between PCO and post‐op flexion angle using a conventional cruciate retaining (CR) prosthesis [3], and there was no impact on post‐operative PROMs using a posterior stabilized (PS) prosthesis, regardless of whether PCOs increased, decreased or remained constant [13].

Post‐operative MPTA, tibial component RA and corrected value of tibial PTA from pre‐op to post‐op showed no significant difference between the two groups. The Robot group had more cases (20 cases) of varus alignment of the tibial component with increased MJG in post‐operative extension than the Conventional group (15 knees).

There were more cases of tibial I/R than tibial E/R in both groups (Table 4); however, Sasaki et al. [38] reported that the upright weight‐bearing tibial anterior‐posterior (AP) axis was positioned in 7.4 of I/R relative to the traditional tibial AP axis. Therefore, we now speculate that the tibial component might be better placed in the E/R position.

Both groups were able to reproduce the pre‐op physiological PTA post‐operatively due to functioning bi‐cruciate ligaments.

NAVIO RA‐BCS TKA achieved a better FJS‐12 score 2 years post‐operatively than conventional BCS TKA [14]. In a multi‐centre study using an image‐based RA arm system (MAKO Surgical Corporation, Stryker), Joo et al. reported that there were significant gradual improvements in PROMs from baseline pre‐operatively to 1–2 years and then to >2 years of follow‐up [24]. In this study, the standard and advanced activities in the 2011 KSS improved in the short post‐op period, demonstrating the benefits of using XR in the RA system.

There was no significant improvement in post‐op flexion angle in the Robot group (Tables 2, 3, 4). Several studies [11, 22, 23, 30, 32, 35, 37, 43] have reported that a post‐op flexion angle is undesirable in conventional BCR TKA (Table 5). A complex surgical technique is one of the reasons for the decrease in the post‐operative flexion angle of BCR TKAs [34]; however, the exact intra‐operative factors necessary to improve the flexion angle after RA‐BCR TKA could not be clarified.

In this study, we determined the following boundaries that MJG from extension to flexion was not constant, but slightly larger, and if possible, LJG in flexion should not exceed 5 mm (Table 6). In the future, we plan to increase the number of cases to determine whether these joint gaps affect post‐operative mid‐ to long‐term PROMs, and to determine ‘cut‐off’ values including the boundaries.

**Table 6:** Intra‐operative joint gap (medial and lateral) in 0° and 90° of flexion in Robot group.

Knee flexion (°)	Medial joint gap (mm)	Lateral joint gap (mm)
0	1.5 (SD 0.8; −0.6 to 4.8)	2.5 (SD 1.1; 0.2–4.9)
90	2.0 (SD 0.7; −0.1 to 4.2)	4.5 (SD 1.5; 0.1–7.1)

*Note*: Mean, standard deviation (SD) and range were provided.

Severely varus knee osteoarthritis is characterized by the presence of osteophytes posterior to the medial femoral condyle and medial tibial plateau. The sizes of the osteophytes depend on the severity of each patient's deformity, and therefore, it is difficult to pre‐operatively predict the post‐operative soft tissue balancing. Katagiri et al. [29] reported that a 5 mm osteotomy of the bone formation enlarges the joint gap by 0.8 mm. In this study, all patients had mild or moderate deformity. Hence, the sizes of the osteophytes were not affected by the post‐operative soft tissue balancing.

The clinical relevance of the present case is that pre‐operative joint line restoration and increasing the lateral PCO using RA techniques prevented the post‐operative flexion contracture and improved standard and advanced activity in the 2011 KSS.

The current study has several limitations. First, the number of cases was small. Second, the post‐op period was short. Third, pre‐ and post‐operative 3DCT measurements were taken in the supine position, so the lower limb alignment under weight‐bearing conditions was not measured, and the exact cartilage thickness and osteotomy volume were not taken into account.

Franceschini et al. [17] revealed that medial meniscus lesions, alone or in association with lateral meniscus lesions, determine a significant increase in the anterior tibial translation compared to knees without meniscus tears. Therefore, the surgeon must keep in mind that the XR prosthesis will have less AP stability than preoperatively due to the lack of meniscus function.

Larger prospective studies with mid‐ and long‐term outcomes are required to further substantiate our findings. We expect that future studies will continue to indicate that RA techniques are superior to conventional techniques in terms of their impact on PROMs. In conclusion, RA‐BCR TKA improved post‐op short‐term PROMs compared to the conventional jig technique.

## AUTHOR CONTRIBUTIONS

Analyzed the data, wrote and edited the paper: Takao Kaneko. Conception of the study: Kosuke Shiga and Shu Yoshizawa. Analyzed the data: Ayakane Yamamoto, Kazuki Amemiya and Masaru Omata.

## CONFLICT OF INTEREST STATEMENT

The authors declare no conflicts of interest.

## ETHICS STATEMENT

The hospital ethics committee approved the study protocol, and patients provided informed consent for participation in the study. All procedures performed in studies involving human participants were in accordance with the ethical standards of the institutional and/or national research committees and with the 1964 Helsinki Declaration and its later amendments or comparable ethical standards. Institutional Review Board approval for the study was provided by Ichinomiya Onsen Hospital, Adult Reconstruction Center (21013). Informed consent was obtained from every participant in the present study.

## Data Availability

There are no available data.
